# College Freshmen Students’ Perspectives on Weight Gain Prevention in the Digital Age: Web-Based Survey

**DOI:** 10.2196/publichealth.7875

**Published:** 2017-10-12

**Authors:** Courtney M Monroe, Gabrielle Turner-McGrievy, Chelsea A Larsen, Karen Magradey, Heather M Brandt, Sara Wilcox, Beth Sundstrom, Delia Smith West

**Affiliations:** ^1^ Arnold School of Public Health, University of South Carolina Columbia, SC United States; ^2^ Department of Communication, College of Charleston Charleston, SC United States

**Keywords:** weight gain prevention, college freshmen, social media

## Abstract

**Background:**

College freshmen are highly vulnerable to experiencing weight gain, and this phenomenon is associated with an increased risk of chronic diseases and mortality in older adulthood. Technology offers an attractive and scalable way to deliver behavioral weight gain prevention interventions for this population. Weight gain prevention programs that harness the appeal and widespread reach of Web-based technologies (electronic health or eHealth) are increasingly being evaluated in college students. Yet, few of these interventions are informed by college students’ perspectives on weight gain prevention and related lifestyle behaviors.

**Objective:**

The objective of this study was to assess college freshmen students’ concern about weight gain and associated topics, as well as their interest in and delivery medium preferences for eHealth programs focused on these topics.

**Methods:**

Web-based surveys that addressed college freshmen students’ (convenience sample of N=50) perspectives on weight gain prevention were administered at the beginning and end of the fall 2015 semester as part of a longitudinal investigation of health-related issues and experiences in first semester college freshmen. Data on weight gain prevention-related concerns and corresponding interest in eHealth programs targeting topics of potential concern, as well as preferred program delivery medium and current technology use were gathered and analyzed using descriptive statistics.

**Results:**

A considerable proportion of the freshmen sample expressed concern about weight gain (74%, 37/50) and both traditional (healthy diet: 86%, 43/50; physical activity: 64%, 32/50) and less frequently addressed (stress: 82%, 41/50; sleep: 74%, 37/50; anxiety and depression: 60%, 30/50) associated topics within the context of behavioral weight gain prevention. The proportion of students who reported interest in eHealth promotion programs targeting these topics was also generally high (ranging from 52% [26/50] for stress management to 70% [35/50] for eating a healthy diet and staying physically active). Email was the most frequently used electronic platform, with 96% (48/50) of students reporting current use of it. Email was also the most frequently cited preferred eHealth delivery platform, with 86% (43/50) of students selecting it. Facebook was preferred by the second greatest proportion of students (40%, 20/50).

**Conclusions:**

Most college freshmen have concerns about an array of weight gain prevention topics and are generally open to the possibility of receiving eHealth interventions designed to address their concerns, preferably via email compared with popular social media platforms. These preliminary findings offer a foundation to build upon when it comes to future descriptive investigations focused on behavioral weight gain prevention among college freshmen in the digital age.

## Introduction

### Background

It is well established that weight gain and the onset of obesity are common in college students [1]. Over one-third of college students were overweight or obese in 2016 [2]. Although both men and women are vulnerable to weight gain across each year of college [3-6], freshmen are at the highest risk for the greatest weight increases [4]. Weight gain during young adulthood is associated with an increased risk of chronic disease and mortality in older adulthood [7-9]. Lifestyle behaviors such as physical inactivity, unhealthy diet, poor sleep habits, and inadequate stress management have been linked to weight gain [10-15]. Behavioral interventions can address these topics and thus potentially attenuate adverse weight gain [16].

Technology represents a particularly attractive and scalable platform for behavioral weight gain prevention interventions in college students. Nearly all (99%) college students regularly use the Internet [17] to access information and socialize with others via nonmobile and mobile devices, especially through Web-based social media platforms. Most college students (80%) report using Web-based social networks (eg, Facebook, Instagram, and Twitter) [17]. Increasingly, weight gain prevention programs that capitalize on the appeal and widespread reach of Web-based technologies (ie, programs that represent one area of the Internet or electronic health [eHealth] interventions) [18] are being evaluated in college students [19-23], and these studies [19-23] have shown mixed findings for effectively promoting maintenance of current weight. Moreover, few of these weight gain prevention interventions [21] are informed by college students’ perspectives on this topic (ie, concerns about weight gain and associated lifestyle behaviors, interest in the specific components that might be included in a comprehensive eHealth weight gain prevention program, and preferred delivery medium). Indeed, findings from a recent systematic review of literature, focused in part on weight gain prevention in young adults, prompted a call to better understand young adults’ attitudes toward engaging in healthy lifestyle behaviors, as well as their weight gain prevention intervention preferences [16]. Such formative information is critical for designing effective technology-based, weight gain prevention interventions for college students, including the subgroup most susceptible to experiencing this phenomenon—freshmen.

Concerns regarding physical inactivity and poor dietary habits among college freshmen have been described previously [24-26], but these studies do not provide a detailed assessment of the range of topics relevant to effective weight gain prevention. Furthermore, a national survey by the American College Health Association [27] reported that two-thirds of college students are interested in receiving information about physical activity and diet, but only a few previous studies have measured interest in [28] and preferred delivery medium [28,29] for receiving an eHealth intervention focused on the promotion of healthy weight [28] or physical activity and diet [29]. One of these studies focused solely on young adult women and weight loss [28], and both studies do not differentiate among popular social media platforms [28,29]. There is a clear need to continue to build upon this small research base and address these topics within the context of weight gain prevention. Doing so will further help guide investigators and practitioners interested in efficiently and effectively addressing college freshmen weight gain via refined, technology-oriented approaches.

### Objective

Thus, the primary purpose of this study was to collectively gauge college freshmen students’ level of concern about weight gain prevention topics, level of interest in and preferred delivery platforms for eHealth programs focused on these topics, and technology use in general and for the purposes of maintaining current weight.

## Methods

### Study Design

Survey data concerning college freshmen students’ perspectives on weight gain prevention were collected as part of a longitudinal investigation focused on health issues and first semester experiences of college freshmen. Specifically, two separate Web-based surveys were administered to a sample of male and female college freshmen at the beginning (baseline) and end (follow-up) of the fall semester.

### Participant Recruitment and Eligibility

A convenience sample of incoming college freshmen was recruited from a large public university in the Southeastern United States during the first 2 weeks of the fall semester via fliers distributed around campus, as well as the direct distribution of fliers in conjunction with verbal invitations in large freshmen classes and at the student union. Individuals had to be self-identified first-year college freshmen with Internet access to participate in the study.

### Procedures

The recruitment fliers provided the directions and link (URL) needed to access the baseline Web-based survey. Before the completion of this survey, participants gave informed consent via a secure website. The survey was available to complete beginning the first day of class (August 20, 2015) and ending on September 2, 2015. Participants received an email 11 weeks later, at the end of the fall semester, asking them to complete the follow-up Web-based survey. The opportunity to complete this follow-up assessment occurred over a 1-month time period (November 4, 2015-November 30, 2015). Participants who completed the Web-based surveys were eligible for a gift card drawing (US $50 drawing at baseline and US $100 drawing at follow-up). The study was approved by the University of South Carolina’s institutional review board.

### Survey Measures

The baseline and follow-up surveys were developed and administered via a secure website (Qualtrics, Provo, Utah). Variables of present interest measured as part of either the baseline survey or follow-up survey are described below.

#### Concern About Weight Gain Prevention and Associated Topics

As part of the follow-up survey, students reported their level of concern about six specific health topics (ie, weight gain, stress management, getting enough sleep, anxiety and depression, eating a healthy diet, and maintaining physical activity during the freshmen year) on a 4-point Likert scale (not concerned, neutral, somewhat concerned, and very concerned).

#### Interest in eHealth Promotion Programs and Preferred Delivery Platform

As part of the follow-up survey, students were asked to respond to the following item: “Please rate how interested you might be in an eHealth promotion program (such as through Facebook, Twitter, Instagram, etc) tailored to college freshmen on these different topics.” Students specifically rated their level of interest on different health topics relevant to weight gain prevention (ie, maintaining a healthy weight, staying physically active, eating healthy on campus, handling stress, depression and anxiety, and adequate sleep) on a 4-point Likert scale (not interested, neutral, somewhat interested, and very interested). Students also responded to the following item on the baseline survey: “If you were to receive health information delivered by social media, which would be your preferred platforms from which to get the health promotion information? Please select up to 3 to indicate your top 3 choices.” Response options to this item included the following: email, Twitter, Facebook, Instagram, Vine, private discussion board or bulletin board or forum, Snapchat, other (specify), and would prefer not to receive health information via social media.

#### Technology Use

Students indicated which popular electronic platforms they used regularly via the baseline survey (ie, email, Twitter, Facebook, Instagram, Vine, private discussion board or bulletin board or forum, Snapchat, other [specify], and do not use any social media or electronic platforms) [30,31].

#### Weight Gain Prevention Practices

##### Self-Weighing

Students reported their frequency of self-weighing on the baseline survey (ie, 1 time/day, 1 time/week, a few times/month, or less often or never).

##### Self-Monitoring of Diet and Physical Activity

Students also responded to items about whether or not they used a smartphone app to track diet, used a smartphone app to track physical activity, and used a wearable physical activity monitoring device on the follow-up survey.

#### Demographics

Demographic data, specifically age, sex, race, and ethnicity, were obtained as part of the baseline survey.

#### Height, Body Weight, and Body Mass Index

Students reported their height and body weight via the baseline Web-based survey. Body mass index was calculated as weight (kg)/height (m^2^).

### Statistical Analyses

Statistical analyses were conducted using the statistical package for the social sciences (SPSS) version 22.0 for Windows (IBM Corp). Descriptive statistics were calculated for all variables. Means and standard deviations (SDs) are presented for continuous variables. Frequencies and percentages are presented for categorical variables. For the items addressing level of concern about specific health topics, very concerned and somewhat concerned response categories were aggregated to highlight the collective proportion for which there was at least some level of concern. Similarly, for the items indicating degree of interest in eHealth promotion programs focused on different topics, very interested and somewhat interested responses were coalesced to highlight the collective proportion who expressed at least some interest. Independent *t* tests and chi-square analyses were used to measure differences in baseline characteristics between study completers and noncompleters. A *P* value of less than .05 was used to determine statistical significance.

## Results

### Sample Characteristics

Fifty-five college freshmen enrolled in the study and completed the baseline survey. Fifty (91%, 50/55) also completed the follow-up survey. Those who completed both surveys averaged 18.3 years of age (SD 0.6) and were predominantly white and normal weight. Two-thirds were women (Table 1). There were no significant differences in any baseline variables between the study completers and noncompleters. Analyses focused on those who completed both surveys and thus provided responses for all variables of present interest.

### Survey Findings

Table 1 summarizes demographic and physical characteristics, reported health concerns, interest in eHealth programs, and weight gain prevention practices among the sample. Three-fourths of students reported being concerned about weight gain during their first college semester, and an even greater proportion reported being concerned about eating a healthy diet (86%, 43/50) and managing stress (82%, 41/50). A low proportion of students reported engaging in empirically validated weight gain prevention strategies [26,32,33] such as weighing oneself at least once a week (22%, 11/50), self-monitoring dietary intake via a smartphone app (12%, 6/50), and self-monitoring physical activity using a smartphone app (24%, 12/50) or wearable device (12%, 6/50). The proportion of students who were at least somewhat interested in receiving an eHealth promotion program focused on different weight gain prevention topics ranged from 52% (26/50) for a program focused on stress management to 70% (35/50) for programs focused on physical activity and healthy eating.

**Table 1:** Demographic and physical characteristics, reported health concerns, interest in electronic health (eHealth) programs, and weight gain prevention practices among college freshmen, fall 2015 semester.

Measure		All (N=50)
Age (years), mean (SD^a^)		18.3 (0.6)
**Sex, n (%)**		
	Female	33 (66)
	Male	17 (34)
**Race, n (%)**		
	White	43 (86)
	African-American	1 (2)
	Asian	3 (6)
	Multiracial	3 (6)
	Hispanic or Latino	2 (4)
Self-reported height, cm, mean (SD)		171.3 (9.1)
Self-reported weight, kg, mean (SD)		63.3 (1.7)
Self-reported BMI^b^, kg/m^2^, mean (SD)		21.5 (2.9)
**Weight status based on self-reported BMI, n (%)**		
	Underweight	7 (14)
	Normal weight	38 (76)
	Overweight	5 (10)
**Self-reported self-weighing, n (%)**		
	1 time/day	1 (2)
	1 time/week	10 (20)
	A few times/month	15 (30)
	Less often or never	24 (48)
Use a smartphone app to track diet, n (%)		6 (12)
Use a smartphone app to track physical activity, n (%)		12 (24)
Use a wearable device to track physical activity, n (%)		6 (12)
**Very or somewhat concerned about, n (%)**		
	Weight gain	37 (74)
	Stress	41 (82)
	Maintaining physical activity	32 (64)
	Healthy diet	43 (86)
	Sleep	37 (74)
	Anxiety and depression	30 (60)
**Very or somewhat interested in eHealth program on, n (%)**		
	Maintaining a healthy weight	34 (68)
	Handling stress	26 (52)
	Staying physically active	35 (70)
	Healthy eating	35 (70)
	Sleep	30 (60)
	Anxiety and depression	27 (54)

^a^SD: standard deviation.

^b^BMI: body mass index.

**Table 2:** College freshmen students’ (N=50) reported current technology use and preferred methods for receiving an eHealth promotion program in their first college semester (fall 2015).

Program delivery method		Currently use platform^a^ n (%)	Prefer eHealth delivery medium^b^ n (%)
Email		48 (96)	43 (86)
Facebook		33 (66)	20 (40)
Twitter		23 (46)	12 (24)
Instagram		34 (68)	10 (20)
Snapchat		38 (76)	8 (16)
Discussion board or bulletin board or forum		1 (2)	3 (6)
Vine		8 (16)	0 (0)
**Other**			
	Tumblr	1 (2)	0 (0)
	Pinterest	1 (2)	0 (0)
	Groupie	1 (2)	0 (0)

^a^Forty-seven (94%, 47/50) students reported using at least one platform in addition to or other than email.

^b^Six (12%, 6/50) students indicated they would not prefer to receive health information via social media.

Table 2 presents results for electronic platforms utilized and preferred electronic methods for receiving a health promotion program. Of note, email was most frequently selected as a preferred platform for receiving health promotion information, with 86% (43/50) of the students identifying it as a preferred platform. Facebook represented the next most frequently selected preferred eHealth platform as it was chosen by 40% (20/50) of the students. Although Snapchat was the second most widely used platform (76%, 38/50) after email (96%, 48/50), it was one of the least preferred mediums for receiving an eHealth promotion program (16%, 8/50).

## Discussion

### Summary of Principal Findings and Comparison With Existing Literature

This formative study preliminarily suggests that a considerable proportion of college freshmen have concerns about weight gain and associated lifestyle behaviors during their first semester and are interested in receiving eHealth programs focused on different weight gain prevention topics. The findings also indicate that the technology platforms most frequently preferred for receiving eHealth programs are email and Facebook.

To our knowledge, this study is the first to provide an assessment of freshmen college students’ level of concern about a range of weight gain prevention topics. The first semester of freshman year has been identified as the period of highest risk for weight gain during the college experience [4], and the level of concern about weight gain during this period is therefore quite relevant to considerations about how to address this issue [16]. It is also the first study to integrate this assessment of concern with a measure of interest in and preferred delivery medium for receiving an eHealth promotion program focused on different weight gain prevention topics.

Our findings are congruent with previous studies that have gauged freshmen college students’ level of concern about weight gain prevention topics [24-26]. For example, de Vos et al found that 50% of the normal weight students and 56% of the overweight students in their sample of 1095 college freshmen reported being concerned about their eating habits during the fall semester [25]. Furthermore, all first-year college students (N=45) in another previous study indicated their concern for eating healthy and being physically active by reporting that the consequences of not doing so were significant enough to try to avoid [24]. In this study, eating a healthy diet concerned the greatest proportion of freshmen relative to other weight gain prevention topics, and the overall concern for weight gain was evident among three-fourths of the students. Despite these concerns, most freshmen reported not practicing regular self-weighing or using technology to track their diet and physical activity, which reflect key behaviors for weight gain prevention [26,32,33]. These findings are consistent with results from a qualitative assessment of first- and second-year college students’ (N=43) weight gain prevention behaviors, which revealed that most students reported weighing themselves infrequently (<1-2 times/month) and not monitoring their physical activity and diet [26].

Nevertheless, in this study, the students’ level of concern for weight gain prevention and associated topics generally paralleled their level of interest in eHealth promotion programs centered on these topics, pointing to their openness to receive guidance and support about maintaining their current weight through popular technology mediums [17,30,31]. However, the gap between the proportion of students who were concerned about stress (82%, 41/50) and those who were interested in an eHealth program targeting stress management (52%, 26/50) was somewhat large. Unlike more traditional weight gain prevention foci (ie, diet and physical activity), perhaps students cannot conceive how stress management can be effectively facilitated via electronic mediums [29], or they may simply prefer to receive stress management guidance through other means (eg, in person). Future research designs should employ more in-depth quantitative and qualitative assessment methods to help elucidate students’ reasoning for their concerns and interests. The majority of the students’ concern about stress is a particularly notable finding as the role of stress in the etiology of obesity is becoming increasingly recognized [34,35]. Yet, few studies in adults, let alone college freshmen, have applied a considerable focus on stress management to help facilitate a healthy weight [36-38]. The present findings suggest that it may be important to consider offering eHealth weight gain prevention programs targeting relevant traditional and less frequently addressed topics, particularly during students’ first college semester when their concern and interest in eHealth support for most weight gain prevention topics are generally aligned.

Given that email was used and preferred as an eHealth delivery method by the highest proportion of students relative to other platforms, researchers and practitioners may want to consider employing this medium when designing electronic-based weight gain prevention programs for first-year college students [29]. These findings are similar to previous studies in other populations [29,39,40]. For instance, Quintilliani et al [29] electronically surveyed 397 college students about their usage patterns of and preferences for potential eHealth delivery channels. They found that 98% of respondents used email, and it was the most frequently cited preferred eHealth channel (86%). Although a distant second, Facebook was the second most frequently preferred eHealth program delivery platform in this study. Initial studies using email and Facebook among college students seeking to maintain a healthy weight or lose weight have shown promise [23,41,42].

A high proportion of students in this study reported using Snapchat (76%, 38/50), which is consistent with a recent market study of 9381 college students [43]. This market study found that most students (58%) open Snapchat first before checking other social media platforms [43]. However, few students in this study selected Snapchat as one of their top preferred eHealth delivery methods. These findings underscore the need to ask specific questions about preference for program delivery rather than assume social usage patterns reflect health promotion preferences. There is a chance that students could not envision how health promotion program components could be delivered by Snapchat [29] and thus selected other platforms by default. To avoid this, perhaps researchers and practitioners seeking to extend applications of these widely used platforms from social networks to health promotion efforts targeted at college freshmen should consider providing concrete examples of how the platforms might be utilized and then asking about preference. Indeed, one of the primary advantages of technology is that it provides the opportunity to tailor the delivery of messages in such a way that the same messages can be distributed via a variety of platforms.

### Study Limitations and Strengths

This study was characterized by several limitations that must be considered when interpreting the findings. The sample size was small and homogeneous (primarily comprising white college students who were of normal weight). Thus, it is unknown whether different response patterns may have emerged for different subgroups of college freshmen. Similarly, it is unknown how a different target outcome (eg, weight loss) may have affected responses in certain subgroups. The sample also comprised self-selected students who may have had more interest in weight gain prevention, other health issues, and technology than those who did not volunteer. Given these factors, it may be premature to generalize the findings broadly. However, these results point to the need for further exploration into these important research questions in a larger, more diverse sample in the different contexts of weight gain prevention, weight loss, and prevention of weight regain after weight loss. Additionally, this study did not inquire about all potential preferred eHealth delivery platforms, which represents another study limitation. Assessing students’ preferences for an even broader array of widely accessible technology delivery mediums (eg, mobile health tools and features) [44], as it relates to weight gain prevention, should be considered in future research. Similarly, this study did not inquire about preferred eHealth delivery platforms for the facilitation of different behavior change strategies (eg, social support, goal-setting, self-monitoring, and feedback) targeting different types of weight gain prevention-related behaviors. Future investigations should address this aspect and consider employing a mixed methods design to better understand the underlying reasoning behind students’ responses.

Despite these limitations, this study was the first to simultaneously assess concern about and interest in receiving eHealth programs via different delivery platforms focused on a range of lifestyle topics relevant to weight gain prevention among college freshmen, expanding the focus from diet and physical activity to other areas associated with weight gain prevention, which might be pertinent to the target population. Unlike previous studies [28,29], this study also differentiated among various social media platforms when assessing students’ preferred eHealth delivery method.

### Conclusions

Overall, this descriptive, formative study preliminary demonstrated that college freshmen have concerns about a range of weight gain prevention-related topics and are generally interested in receiving eHealth promotion programs focused on these topics. The greatest majority of students selected a traditional medium (ie, email) as their preferred method for receiving an eHealth program, although a substantial proportion also preferred Facebook. This study fully opens the conversation on an impactful and under-researched area, yielding findings that provide a foundation to build upon when it comes to future descriptive investigations focused on behavioral weight gain prevention among college freshmen in the digital age. Ultimately, such studies may help inform the design of future, far-reaching technology-based weight gain prevention interventions that have the potential to enhance program receptiveness, exposure, engagement, and effectiveness for college freshmen.

## Abbreviations

BMI: body mass index
eHealth: electronic health
SD: standard deviation
SPSS: statistical package for the social sciences
